# Self-Reported Patient and Provider Satisfaction With Neurology Telemedicine Visits After Rapid Telemedicine Implementation in an Urban Academic Center: Cross-Sectional Survey

**DOI:** 10.2196/53491

**Published:** 2024-10-30

**Authors:** Noah Robertson, Maryam J Syed, Bowen Song, Arshdeep Kaur, Janaki G Patel, Rohit Marawar, Maysaa Basha, Deepti Zutshi

**Affiliations:** 1 Department of Neurology Wayne State University School of Medicine Detroit, MI United States

**Keywords:** telemedicine, telehealth, neurology, eHealth, teleneurology, patient experience, patient satisfaction

## Abstract

**Background:**

Many clinics and health systems implemented telemedicine appointment services out of necessity due to the COVID-19 pandemic.

**Objective:**

Our objective was to evaluate patient and general provider satisfaction with neurology telemedicine implementation at an urban academic medical center.

**Methods:**

Patients who had completed 1 or more teleneurology visits from April 1 to December 31, 2020, were asked to complete a survey regarding their demographic information and satisfaction with teleneurology visits. Providers of all specialties within the same hospital system were given a different survey to gather their experiences of providing telemedicine care.

**Results:**

Of the estimated 1500 patients who had completed a teleneurology visit within the given timeframe, 117 (7.8%) consented to complete the survey. Of these 117 respondents, most appointments were regarding epilepsy (n=59, 50.4%), followed by multiple sclerosis (n=33, 28.2%) and neuroimmunology (n=7, 6%). Overall, 74.4% (n=87) of patients rated their experience as 8 out of 10 or higher, with 10 being the highest satisfaction. Furthermore, 75.2% (n=88) of patients reported missing an appointment in the previous year due to transportation issues and thought telemedicine was more convenient instead. A significant relationship between racial or ethnic group and comfort sharing private information was found (*P*<.001), with 52% (26/50) of Black patients reporting that an office visit is better, compared to 25% (14/52) of non-Black patients. The provider survey gathered 40 responses, with 75% (n=30) of providers agreeing that virtual visits are a valuable tool for patient care and 80% (n=32) reporting few to no technical issues. The majority of provider respondents were physicians on faculty or staff (n=21, 52%), followed by residents or fellows (n=15, 38%) and nurse practitioners or physician assistants (n=4, 10%). Of the specialties represented, 15 (38%) of the providers were in neurology.

**Conclusions:**

Our study found adequate satisfaction among patients and providers regarding telemedicine implementation and its utility for patient care in a diverse urban population. Additionally, while access to technology and technology literacy are barriers to telemedical care, a substantial majority of patients who responded to the survey had access to devices (101/117, 86.3%) and were able to connect with few to no technological difficulties (84/117, 71.8%). One area identified by patients in need of improvement was comfortability in communicating via telemedicine with their providers. Furthermore, while providers agreed that telemedicine is a useful tool for patient care, it limits their ability to perform physical exams. More research and quality studies are needed to further appreciate and support the expansion of telemedical care into underserved and rural populations, especially in the area of subspecialty neurological care.

## Introduction

Telemedicine refers to the ability to use communication technologies such as the telephone or video chat to remotely manage health care. Telemedicine in neurology, or teleneurology, was first introduced in the 1990s for the treatment of Parkinson disease [1]. Over time, it has developed into other uses, such as allowing patients to gain fast access to stroke specialists to facilitate acute treatments [2,3]. Despite hesitance to adopt teleneurology due to concerns about reimbursement, privacy, and connecting with patients, the COVID-19 pandemic has forced health care providers to adapt and continue providing quality care for patients.

After being declared a pandemic by the World Health Organization (WHO) in March 2020, COVID-19 prompted nationwide shutdowns of all nonessential facilities. Ambulatory clinics experienced a high demand to create an alternative and effective platform to continuously provide high-quality patient care during the pandemic [4].

With the need to limit viral transmission, telemedicine is an excellent way that patients can still receive quality health care while avoiding contact with others, especially health care workers who may have been exposed to COVID-19. With patient services being limited, the relaxation of telemedicine rules and regulations, and insurance companies beginning to reimburse for telemedicine care, there was an opportunity for hospitals to start implementing telehealth services [5,6]. Our objective was to determine the satisfaction of patients and health care providers who had used telemedicine services at least once during the 2020-2021 pandemic period. Our secondary objective was to determine the challenges patients and providers in our Detroit-based community faced when using telemedicine and report on the different aspects of their user experience compared to in-person visits.

## Methods

### Implementation and Study Design

This study took place at the Detroit Medical Center (DMC) in Detroit, Michigan. In response to the COVID-19 pandemic, there was a rapid implementation and transition to telemedicine appointments in the outpatient neurology clinic at the DMC from March 18, 2020, when clinics initially shut down, until April 8, 2020, when telemedicine was initiated. This practice serves all patients in neurology, including subspecialties in general neurology, multiple sclerosis, vascular neurology, neuromuscular medicine, epilepsy, movement disorders, neurobehavior and cognitive disorders, and headache. The conversion of all clinics to telemedicine was conducted as part of routine clinical care within the DMC. This patient population had never been exposed to teleneurology at the DMC prior to the implementation during the COVID-19 pandemic. The study’s goal was to determine patient opinion regarding the implementation of telemedicine as well as provider satisfaction with the rapid implementation of telemedicine throughout metro Detroit.

### Participant Selection

Patients who had at least 1 teleneurology clinic visit from April 1 to December 31, 2020, were asked to complete the survey when they returned to in-person clinic visits. Survey collection occurred from September 2020 to January 2021. All patients, aged 18 years or older, who had a telemedicine clinic evaluation within those dates received the survey after written consent was given. All providers that conducted telemedicine virtual visits after the start of the pandemic received the provider survey. Provider satisfaction surveys were distributed to the Wayne Health practice group, affiliated with the Wayne State University School of Medicine; the DMC Graduate Medical Education office for residents and fellows; and the DMC medical staff organization, which includes staff at the adult central campus hospitals and Children’s Hospital of Michigan. This includes all level of residents, fellows, nurse practitioners, physician assistants, and physicians. Providers who did not participate in a telemedicine consultation were asked to not answer the survey.

### Patient Survey

The patient surveys were adapted with permission from the Massachusetts’s General Hospital Telehealth Virtual Visit Patient survey [7]. Changes to the survey were made, focusing on the COVID-19 pandemic and infrastructure for telemedicine at our institution. We added demographic questions about age, insurance, transportation requirements, and telehealth experiences, and the layout of the survey was reorganized to reduce the number of printed pages patients had to complete. A Likert satisfaction scale was used to assess the patients’ opinions on the quality of care they received, communication with their provider, technology quality, efficiency, convenience, and their acceptance and willingness to use telemedicine in the future. The surveys were administered to patients in a paper format to be self-completed. Because the survey was administered when the patient returned for an in-person visit, there was no contact or follow-up outside of the appointment regarding the survey.

The technical component of the survey asked questions focusing on where they accessed telemedical care, what device type they used, and the connection type (Wi-Fi, cellular data, etc). Survey questions regarding the virtual visit itself were answered with options of “Yes, Definitely,” “Yes, Somewhat,” “No,” and “Don’t Know.” The second section asked patients about their provider interaction and communication quality differences between in-person versus telehealth visits. The third portion of the patient survey asked patients to compare different types of visits with the options “Virtual Visit is Better,” “Office Visit is Better,” “No Difference,” and “Does Not Apply to Me.” This section finished with a question regarding whether the patient would recommend a virtual visit to family and friends (Section S1 in Multimedia Appendix 1).

### Provider Survey

The provider surveys were adapted from the Massachusetts’s General Hospital Telehealth Virtual Visit Patient survey [7]. The survey was adapted to a web-based version using our institutional REDCap (Research Electronic Data Capture; Vanderbilt University) site and distributed as an email in September 2021 to all specialty providers, including those in surgery, medicine, and pediatrics [8]. Providers received follow-up reminder emails, and the survey was closed after 3 months. The questions asked were regarding telehealth services provided during the COVID-19 pandemic, with the intention of determining the quality of communication with patients, the technical quality, convenience, and the willingness to incorporate telemedicine into their practice as a permanent feature.

The first portion of the provider survey asked questions about background information such as age, specialty, level of training, and their current experience with virtual visits. Following that, providers were surveyed on a 5-point Likert scale with the options “Strongly agree,” “Somewhat agree,” “Neither agree nor disagree,” “Somewhat disagree,” and “Strongly disagree” to answer more specific questions about their feelings regarding virtual visits (Section S2 in Multimedia Appendix 1).

### Ethical Considerations

The Wayne State University Institutional Review Board approved human participant research that can maintain remote study interventions under protocol IRB-20-05-2244. All patients and providers had to consent to participate in the survey. All data received from the survey have been anonymized. Patients and providers were not compensated for their participation in the survey.

### Data Collection and Analysis

The patient survey responses were entered manually from the paper format into the REDCap database. Data were then exported from REDCap into Microsoft Excel, and descriptive statistical analysis was performed. Chi-square tests crossing survey results with patient demographics were performed to look for statistical significance.

## Results

### Patient Demographics and Technical Results

Within the examined time period, it is estimated that teleneurology visits were provided to around 1500 patients within the neurology clinic. In total, 117 (7.8%) patients consented to complete part or all of the survey (Table 1). Of the 117 survey respondents, 87.2% (n=102) were aged 18 to 64 years, and 6.8% (n=8) were aged 65 years and older. The majority of patients identified as female (n=78, 66.7%). The most common ethnic or racial group was Black (n=53, 45.5%), followed by White (n=43, 36.8%) and then Hispanic (n=5, 4.3%). Most patients reported an education level of completing high school or higher (n=96, 82.1%). Six neurology subspecialties were represented, with most patients being seen in epilepsy (n=59, 50.4%) and multiple sclerosis (n=33, 28.2%) clinics.

A total of 84 (71.8%) of the patients’ telemedicine visits were follow-ups. Audio or video connection was the most common type of visit (n=60, 51.3%), followed by audio- or telephone-only visits (n=45, 38.5%); the rest did not respond to the survey question. Furthermore, 91 (77.8%) patients reported that they had to miss an appointment in the last year before the COVID-19 pandemic due to issues with transportation. Two-thirds (n=74, 63.2%) of patients relied on transportation from family and friends (n=56, 47.9%), medical transportation (n=12, 10.3%), or public transportation (n=6, 5.1%) in the year prior to their teleneurology visits.

Finally, 75.2% (n=88) of patients used their own cellphones to conduct their teleneurology visit. Patients most commonly used a Wi-Fi network (n=64, 54.7%) or cellular data (n=33, 28.2%) to connect to their visits. Only 20 (17.1%) reported issues during those visits, mostly with connection issues or audio or visual connection issues. Despite these issues, 59.8% (n=70) noted they were still able to complete their visit.

**Table 1:** Demographics of patient survey respondents.

Demographics			Patients (n=117), n (%)
**Age group (years)**			
	18-45	59 (50.4)	
	46-64	43 (36.8)	
	≥65	8 (6.8)	
	No response	7 (6)	
**Racial or ethnic group**			
	White	43 (36.8)	
	Black	53 (45.5)	
	Hispanic	5 (4.3)	
	Other	4 (3.4)	
	Prefer not to answer	4 (3.4)	
	No response	8 (6.8)	
**Education Level**			
	Some high school	15 (12.8)	
	Completed high school or GED^a^	27 (23.1)	
	Some college	43 (36.8)	
	Bachelor’s degree	16 (13.7)	
	Some postgraduate college	3 (2.6)	
	Master’s degree	5 (4.3)	
	Professional degree (PhD, law, medical, etc)	2 (1.7)	
	No response	6 (5.1)	
**Disease Specialty**			
	Epilepsy	59 (50.4)	
	Multiple sclerosis	33 (28.2)	
	Neuroimmunology	7 (6)	
	Movement	5 (4.3)	
	Headache	3 (2.6)	
	General	2 (1.7)	
	No response	8 (6.8)	

^a^GED: General Educational Development.

### Patient Satisfaction

Overall, 87 (74.4%) patients rated their experience with their teleneurology visit as 8 out of 10 or higher, with 10 being the “best visit.” Moreover, 88 (75.2%) patients stated that they would recommend a virtual visit to their family and friends. A substantial majority of patients reported satisfaction with the time spent with their provider. In terms of convenience, 42.2% (n=49) of patients noted that a virtual visit was better for finding time for appointments, and 49.1% (n=57) felt time saved from traveling was better with virtual appointments (Multimedia Appendix 2). Despite satisfaction with virtual visits, 58.1% (n=68) of patients felt that office visits grant better ability to show their physician a physical problem, and 37.6% (n=44) stated that office visits were better for a personal connection with their physician. A significant relationship between racial or ethnic group and comfort sharing private info was found (*P*<.001), with 52% (26/50) of Black patients reporting that an office visit was better, compared to 25% (14/52) of non-Black patients.

### Provider Demographics and Technical Results

A total of 40 providers responded to the provider survey, 4 (10%) of which were nurse practitioner or physician assistants, 21 (52%) were physicians, and 15 (38%) were residents or fellows (Table 2). Providers were mostly female (n=26, 65%) and between the ages 30-39 years (n=19, 48%). Nearly half (n=19, 48%) practiced in a setting that is 75% to 100% outpatient.

At the time of the survey, 13 (32%) providers indicated that they had completed over 100 virtual visits. A fifth (n=8, 20%) of the providers performed virtual visits exclusively with audio and video input, whereas 13 (32%) of them indicated that 75% or more of their virtual visits were via audio or telephone only. About half (n=23, 58%) of the providers exclusively conducted virtual visits at their clinic or office, and the other half (n=17, 42%) preferred to work purely from home or a mix between home and office.

Overall, 80% (n=32) of providers reported little or no technical issues. The most common applications to connect were the Doximity dialer application (n=13, 32%) and Zoom (Zoom Video Communications; n=9, 22%). A total of 75% (n=30) of providers described the processes of setting up their telemedicine station and acclimating to their application of choice as easy, and 48% (n=19) of them felt that after 1 or 2 virtual visits, they were comfortable with the technology. The top 3 problems encountered by providers during their virtual visits were issues with remaining connected, hearing or being heard, and seeing or being seen.

**Table 2:** Demographics of provider survey respondents.

Demographics			Physicians (n=40), n (%)
**Age group (years)**			
	<30	3 (8)	
	30-39	19 (48)	
	40-59	10 (25)	
	>60	8 (20)	
**Provider type**			
	Nurse practitioner or physician assistant	4 (10)	
	Physician on faculty or staff	21 (52)	
	Resident or fellow physician	15 (38)	
**Specialty**			
	General pediatrics or pediatric subspecialty	4 (10)	
	Internal medicine or internal medicine subspecialty	3 (8)	
	Neurology	15 (38)	
	Surgery or surgical subspecialty	6 (15)	
	Other	12 (30)	

### Provider Satisfaction

**Figure 1:**
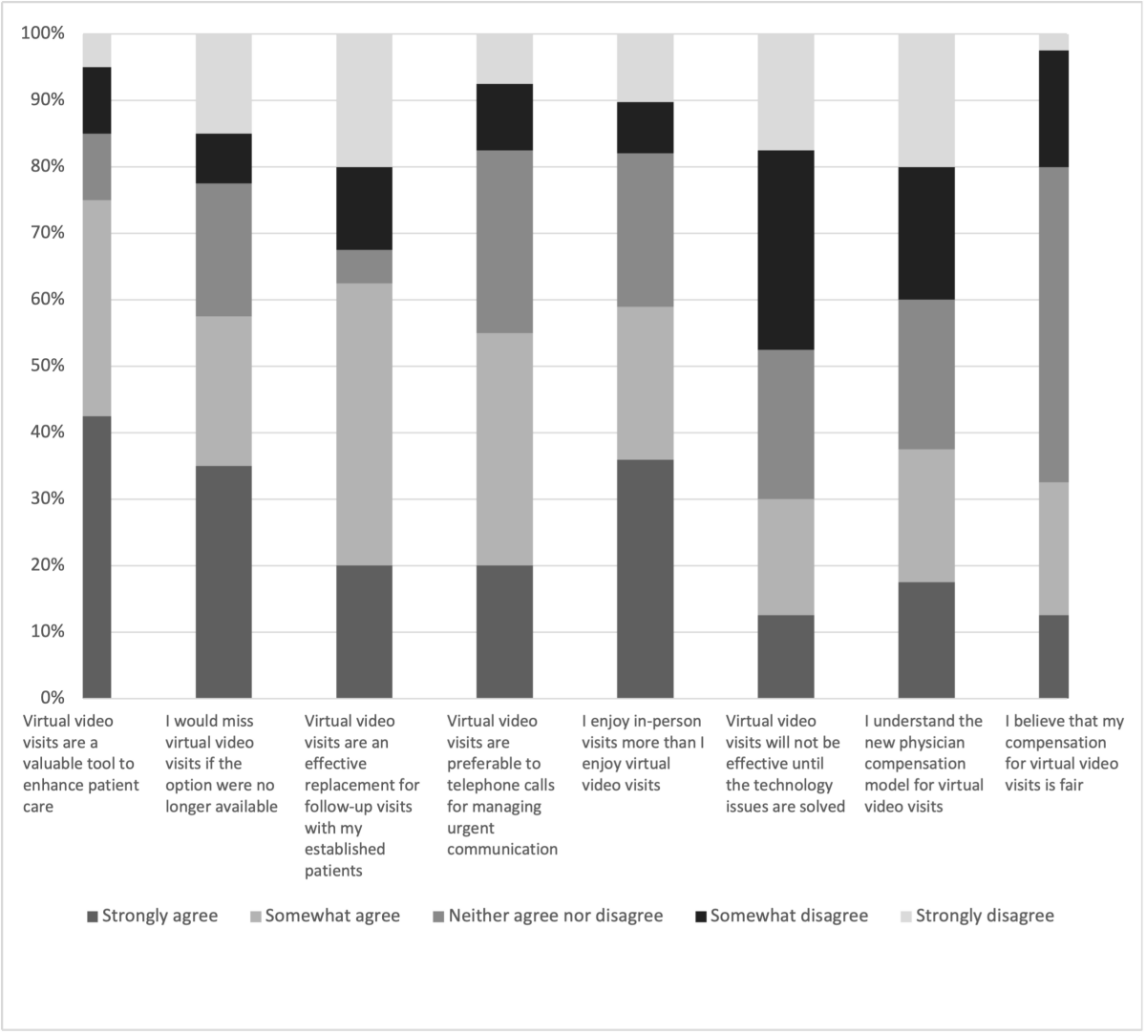
Provider agreement on opinions about virtual audio and/or video visits. Provider-reported agreement on opinions regarding virtual visits, graded on a 5-point Likert scale. A web-based survey was sent in September 2021 to providers of all specialties including surgery, medicine, and pediatrics that are affiliated with Wayne Health practice group, Wayne State University School of Medicine, the Detroit Medical Center (DMC) Graduate Medical Education office for residents and fellows, and DMC medical staff organization. Providers were asked to only complete the survey if they had previously completed a telemedicine visit, and there was a 3-month window for responses. In total, 40 providers responded to the survey. Results are given in percentages (%).

Overall, 75% (n=30) of providers agreed that virtual visits are a valuable tool to enhance patient care, and 70% (n=28) would recommend a virtual visit to their family members or friends. Furthermore, 25 (62%) respondents agreed that telemedicine offers an effective replacement for follow-up appointments, and in terms of efficiency, 42% (n=17) thought virtual visits were better (Multimedia Appendix 3). However, the majority of providers (23/40, 57%) enjoyed in-person visits more than virtual ones, and they overwhelmingly agreed that office visits were better for seeing physical problems (Figure 1). Of the 15 trainee physicians who responded, 9 (60%) agree that they would miss video visits if they were no longer an option and 3 (20%) were indifferent, whereas 13 (62%) of the 21 physicians on faculty would miss video visits and 3 (14%) reported indifference. Interestingly, while half (20/40, 50%) stated that office visits were better for developing a personal connection with their patients, 42% (n=17) noted they felt no difference with the appointment modalities. Lastly, fewer than 40% (n=15) of providers agreed that they understood the compensation model for virtual video visits.

## Discussion

Our study reviewed patient satisfaction with rapid implementation of telemedicine in a neurology clinic in an urban city population. We found that the majority of patients were satisfied with their telemedicine visits. Although inequalities regarding electronic device access and technology literacy exist [9], we found that almost all patients had access to technology and internet to connect with providers with few issues. We also found that providers who had never before seen patients with telemedicine found it to be a useful tool to provide care for patients.

In our study, patients have expressed satisfaction with the implementation of telemedicine, with the average rating equaling to 8.7 out of 10. This high level of satisfaction is similar to other studies, with one paper reporting a 99% patient satisfaction rate among patients with chronic neurological disorders [10]. It has been shown that patients agree that they would recommend teleneurology visits to friends and family, as well as consider virtual visits in the future at high rates [11-13].

The quality of communication between patient and provider was lower than other areas. Regarding feeling a personal connection with their physician, being comfortable sharing personal information, and showing their clinician a physical problem, more patients agreed that an office visit is better, or that they felt no difference. Differences between racial or ethnic groups may be related to previous experiences of discrimination and racism, which can create mistrust between patients and providers [14,15]. This may be further compounded in patients with neurological diseases where more complications and progression of certain conditions may require clear communication [16]. Effective communication between patients and their physicians is correlated with better patient outcomes [17,18]. A solution for this could include modules or courses to teach providers ways to better build rapport with patients when communicating electronically, which have been shown to increase communication scores [19,20].

Telemedicine proved particularly advantageous to our cohort when examining transportation, of which almost 80% of respondents noted issues, causing them to miss an appointment within the year prior to the pandemic. This finding is notable, especially in the context of our survey’s location because Detroit is a city with limited public transportation, and it has been shown that urban Detroit men and women have to travel longer distances for daily activities compared to nearby suburban residents [21]. Our cohort had more patients who reported the convenience of scheduling the telemedicine visit and that travel time was better for telemedicine, which highlights the usefulness of telemedicine in reaching patients who may not have access to easy transportation to clinic visits.

Lastly, while it has been shown that low-income households are less likely to have devices capable of internet services [9], 86.3% (101/117) of patients in our study had their own personal device to connect to their visit, and 71.8% (84/117) had no technical issues. Although it is promising that the majority had devices, there must still be awareness of barriers to telemedicine care and effort toward expanding access as well as improving the quality of visits.

All in all, providers were very flexible and effective in their adoption of telemedicine. They appreciated the added flexibility of virtual appointments, and a substantial majority (75%) agreed that virtual visits were an important tool to enhance patient care. Similar results have been observed among other physicians that experienced a rapid implementation of telemedicine [22,23]. Providers in our study almost unanimously agreed that an office visit was preferred when it came to the ability to see a physical problem. This is relevant for specialties like neurology, pediatrics, and dermatology where physical exams are important for localizing issues and ruling out diagnoses. This is most likely the reason why virtual visits were more likely to be acceptable for return or follow-up visits [22,24], and why half of our providers agreed that video visits were preferred to telephone calls.

Interestingly, fewer than 40% of providers agreed that they understood the telemedicine compensation model implemented during the pandemic. Reimbursement rates were made equivalent to in-person visits during the pandemic [25]. However, these rates for audio- or telemedicine ended in May 2023, as they were based on policies that were in place to help decrease the spread of COVID-19 [26]. An extension of said policies and reimbursement schemes past the pandemic would be beneficial to allow more time for research to determine outcomes of telemedicine and to continue further improvements in expansion and technology for telemedicine services. If more physicians understood how they could be compensated for their telemedicine services, it could also create more opportunities to expand care into underserved communities and rural population centers, which are reported to be at risk to have a shortage of specialty providers in the future [27,28].

One source of bias within our study is recall bias, because patients were responding from their memory of teleneurology appointments that took place months earlier. Moreover, within that time period, it is possible patients already had an in-person office visit influencing their original opinions of their previous virtual visit. Another limitation is the small sample size (117 patients and 40 providers), which prevented statistical analysis beyond chi-square tests and descriptive statistics. This was compounded by the wide array of answer choices and the answer distribution among them. The number of “no answers” within the completed patient surveys could be due to the fact that the survey was self-administered with no required entry fields, so the patients had the ability to choose which questions they wanted to answer. The observed response rate of 7.8% (117 responses out of 1500 patients seen in clinic) could be due to several factors, such as patient refusal, patients reporting inadequate recollection of their previous telemedicine visit, lack of time to complete survey in a busy clinical practice, and lack of incentive. Furthermore, many patients that completed teleneurology visits may not have returned to the clinic for an in-person visit within the 4-month patient survey period. There is a potential selection bias influencing the results, because only patients who completed at least 1 telemedicine visit were surveyed, which means those without access that depended on in-person visits are not represented. Patients who agreed to the survey may have done so due to overly positive experiences, overly negative experiences, or for reasons unrelated to health care such as logistics, which could influence them to answer to the extremes in one way or another. This selection bias can apply to the physician survey as well, as not all specialties are equally represented, possibly due to the fact that telemedicine use is lower for some subspecialties such as orthopedic surgery or urology when compared to higher usage specialties like endocrinology or behavioral health [29]. Lastly, we note that the small patient sample size restricts the ability to generalize the results to the background population sampled as well as other neurology clinics that offer telemedicine visits.

With the end of the public health emergency on May 12, 2023, many health care allowances that were created were reversed. However, the impact that telemedicine had on patient care was significant across the country and in many different types of communities. As such, the continued use of telemedicine and expansion to more rural and underserved communities is vital to the health of the US population.

## Appendix

Multimedia Appendix 1 The patient and provider surveys that were administered for the study.

Multimedia Appendix 2 Patients from the Detroit Medical Center in Detroit, Michigan, reported their preference for in-person office visits or virtual audio and/or video visits based on certain determining factors. These results are from a survey administered to patients, aged 18 years or older, who had at least 1 teleneurology visit between April 1 and December 31, 2020, and who had provided written consent for the survey. Surveys were collected between September 2020 and January 2021. In total, 117 patients participated in the survey Results are given in percent (%).

Multimedia Appendix 3 Provider-reported appointment preferences based on factors that influence appointment satisfaction. A web-based survey was sent in September 2021 to providers of all specialties including surgery, medicine, and pediatrics that are affiliated with the Wayne Health practice group, Wayne State University School of Medicine, the Detroit Medical Center (DMC) Graduate Medical Education office for residents and fellows, and DMC medical staff organization. Providers were asked to only complete the survey if they had previously completed a telemedicine visit, and there was a 3-month window for responses. In total, 40 providers responded to the survey. Results are given in percent (%).

## Abbreviations

DMC: Detroit Medical Center
REDCap: Research Electronic Data Capture
WHO: World Health Organization

## Data Availability

Anonymized data are available upon request from any qualified investigator.
