# Supplementary material for: Self-Reported Patient and Provider Satisfaction With Neurology Telemedicine Visits After Rapid Telemedicine Implementation in an Urban Academic Center: Cross-Sectional Survey
Source: JMIR Form Res. 2024 Oct 30;8:e53491. doi: 10.2196/53491 (PMC11561435; doi:10.2196/53491)
Supplement: Multimedia Appendix 1 [file formative_v8i1e53491_app1.docx]

**Section S1.** PAST-COVID patient survey, 3 pages.


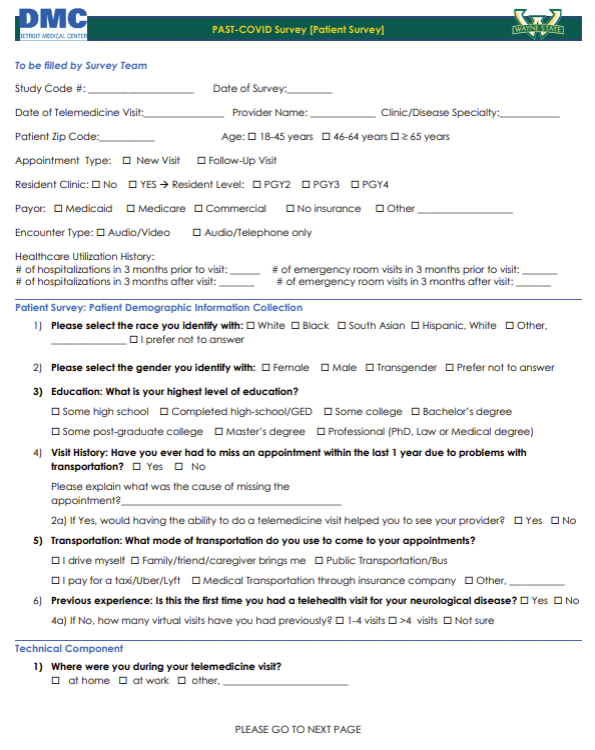


**Figure S1.** Page 1 of patient survey, including demographics and start of technical component.


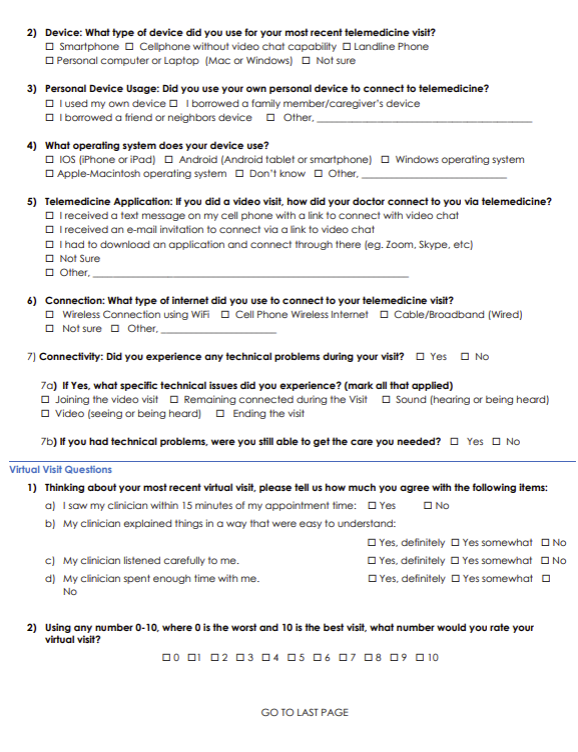


**Figure S2.** Page 2 of the patient survey, including the technical component and visit satisfaction questions.


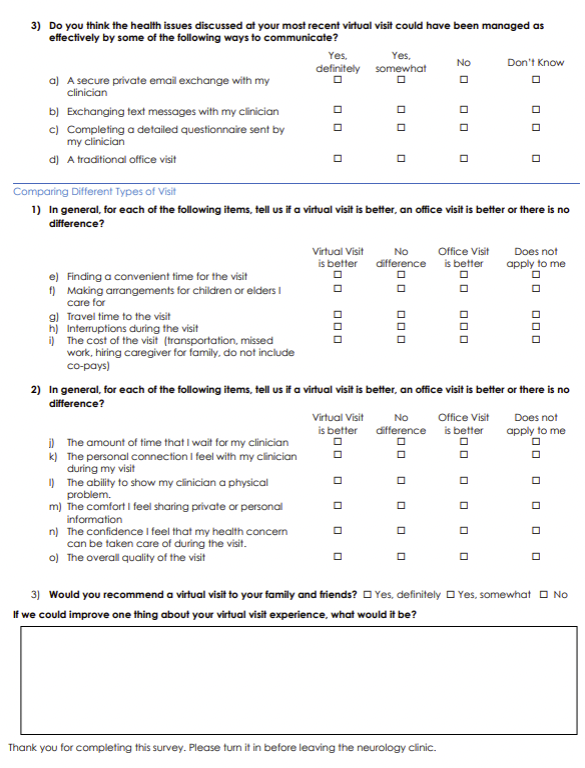


**Figure S3.** Page 3 of the patient survey, including satisfaction questions, questions comparing office visits to virtual visits, and free response box.

**Section S2.** PAST-COVID provider survey, 5 pages.


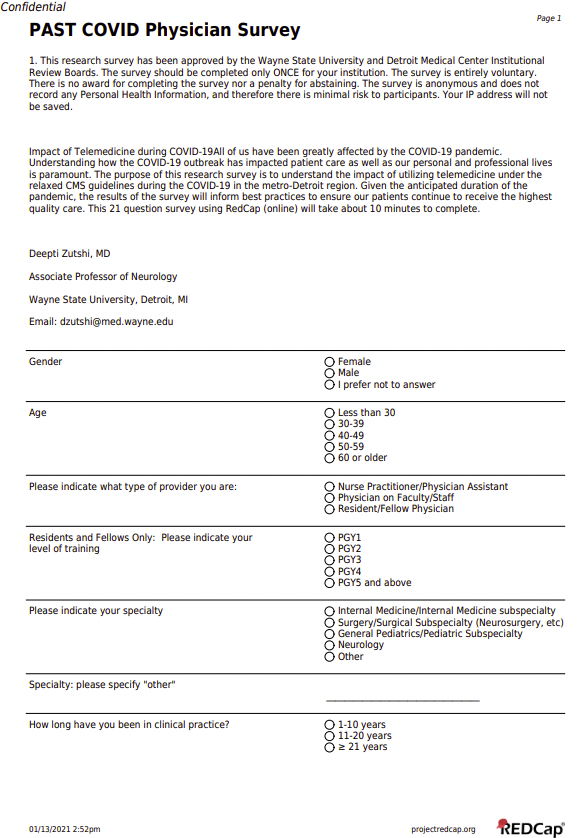


**Figure S4.** Page 1 of the provider survey, with questions mainly on demographics.


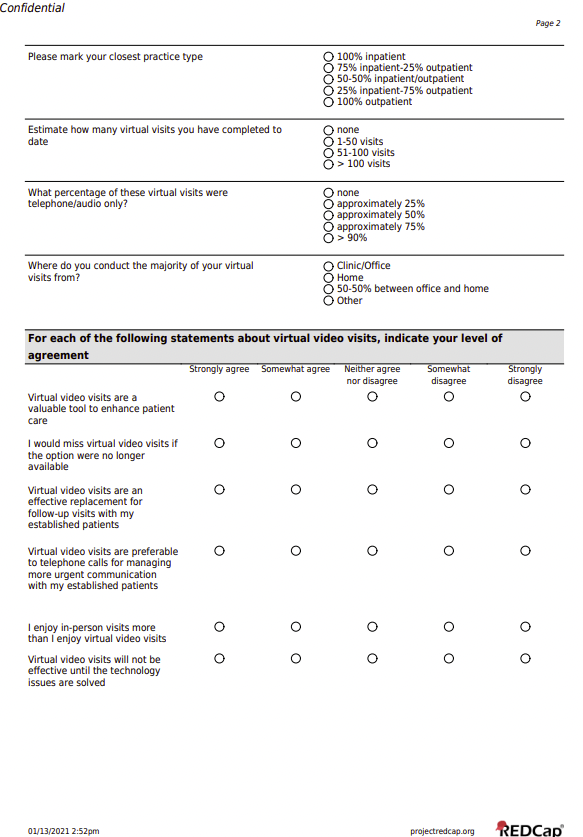


**Figure S5.** Page 2 of the provider survey, finishing demographic questions and 5-point Likert scale questions about virtual video visits.


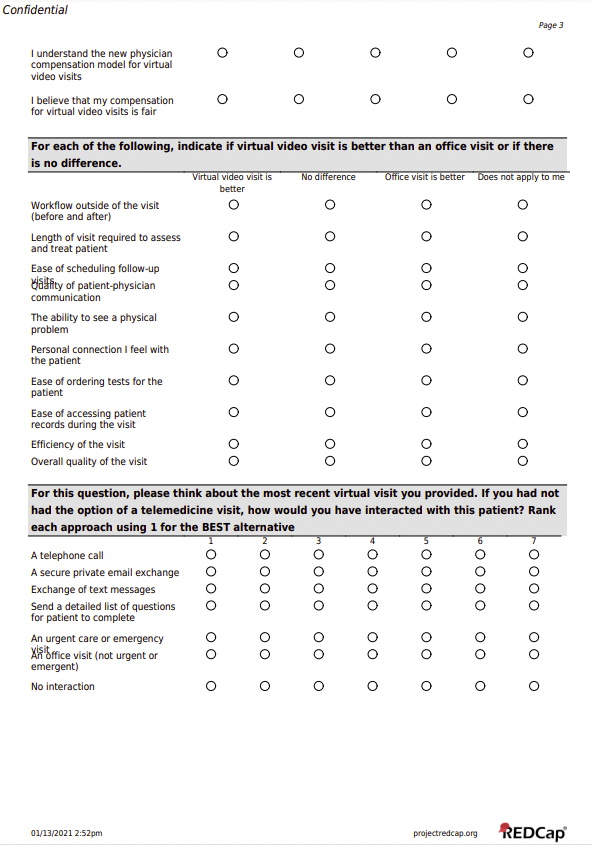


**Figure S6.** Page 3 of the provider survey, with final questions about virtual video visits, questions regarding visit modality preference, and virtual contact method preferences.


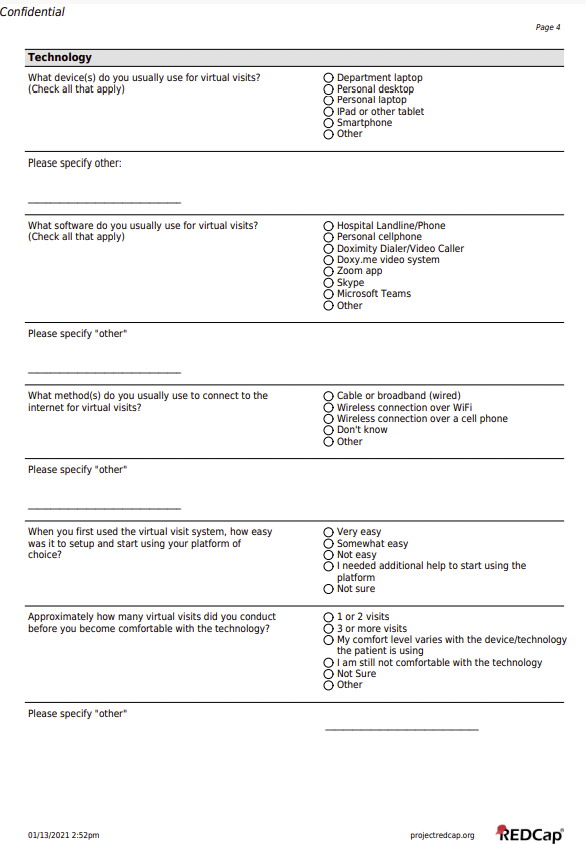


**Figure S7.** Page 4 of the provider survey, the technical component to understand how virtual visits were conducted.


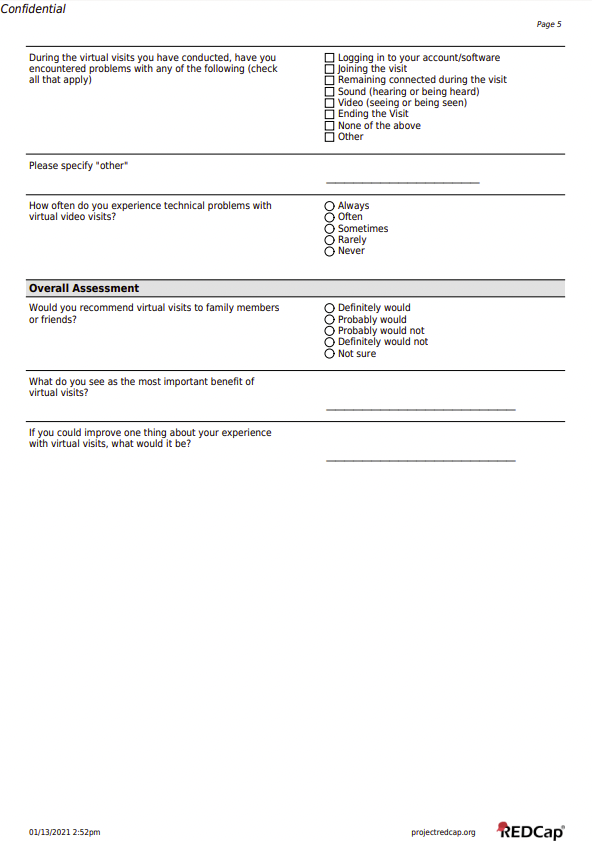


**Figure S9.** Page 5 of the provider survey, the final questions of the technical component, and the overall provider satisfaction assessment.
