# Supplementary figures and images for: Self-Reported Patient and Provider Satisfaction With Neurology Telemedicine Visits After Rapid Telemedicine Implementation in an Urban Academic Center: Cross-Sectional Survey
Source: JMIR Form Res. 2024 Oct 30;8:e53491. doi: 10.2196/53491 (PMC11561435; doi:10.2196/53491)

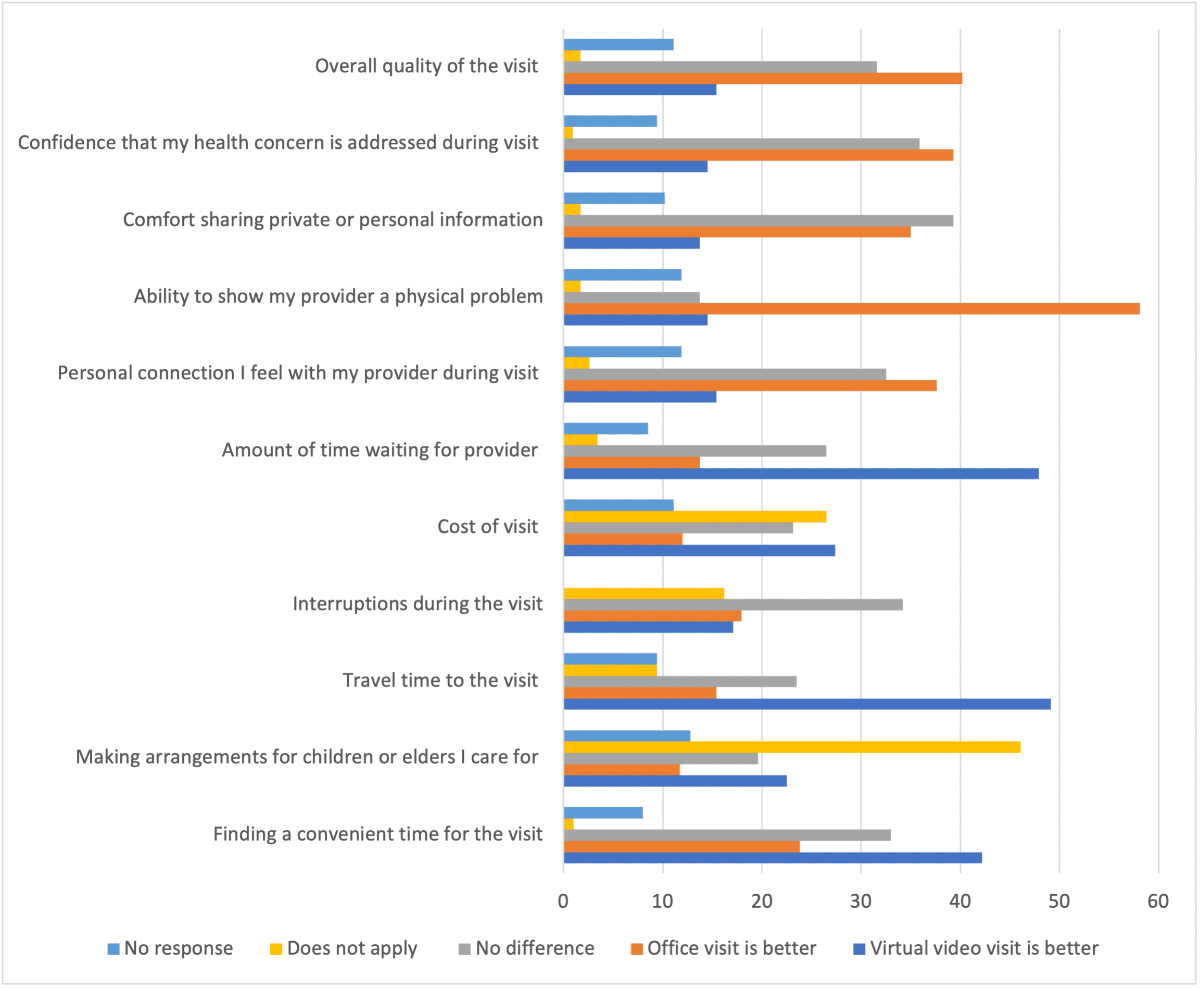

Supplement: Multimedia Appendix 2 [file formative_v8i1e53491_app2.png]

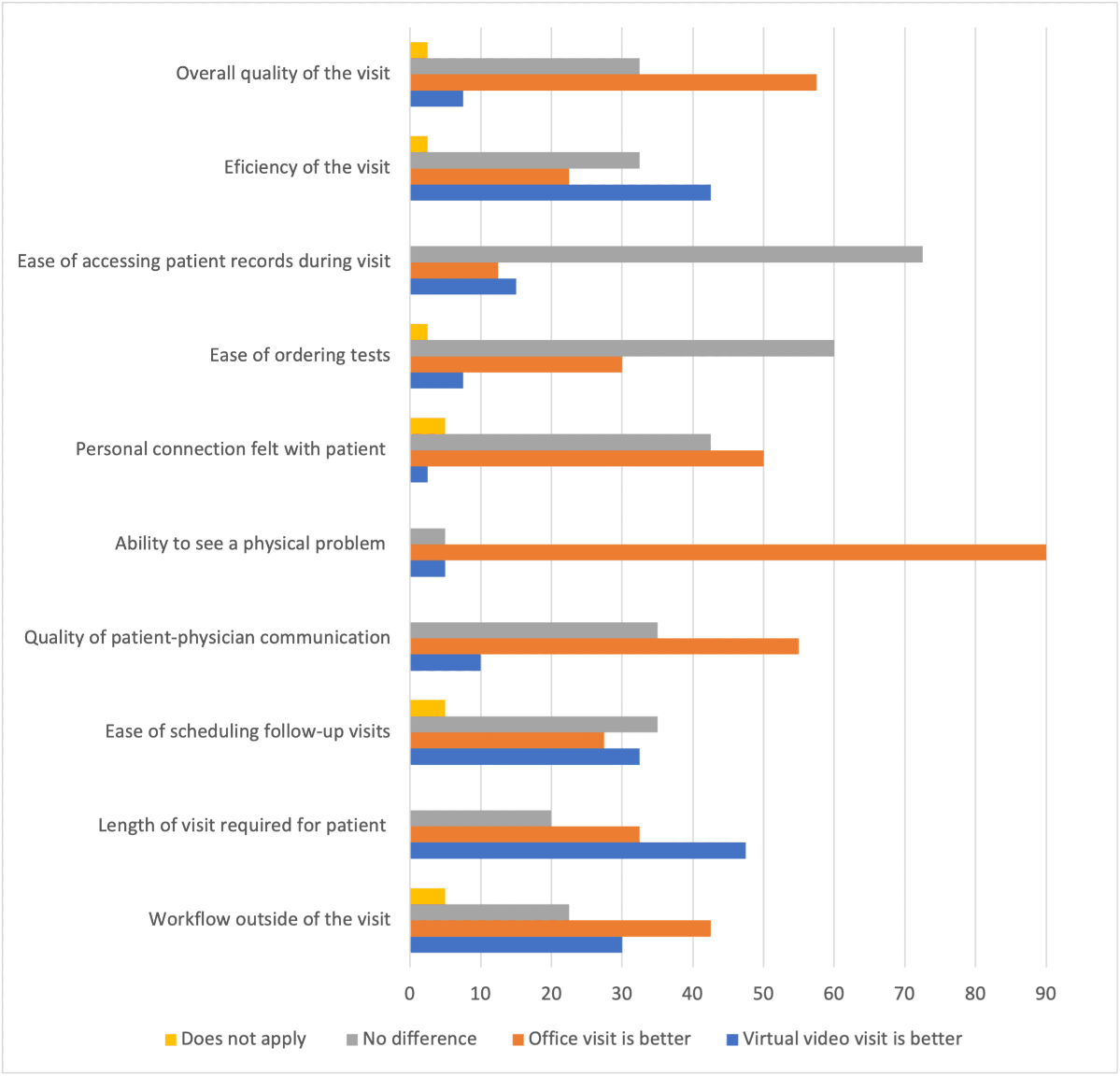

Supplement: Multimedia Appendix 3 [file formative_v8i1e53491_app3.png]
